# A Python GUI for integrated regional analysis of GWAS-identified QTLs

**DOI:** 10.1186/s44342-026-00091-6

**Published:** 2026-11-10

**Authors:** Partha Talukdar, Michael Martin

**Affiliations:** 1 Guy’s and St. Thomas’ NHS Foundation Trust, London, UK; 2 School of Biological Sciences, University of Aberdeen, Aberdeen, UK

**Keywords:** Graphical user interface, GWAS, Linkage disequilibrium, Quantitative trait locus, SNP, Genotype–phenotype analysis

## Abstract

Genome-wide association studies (GWAS) identify genomic regions associated with complex traits, but interpreting these regions requires further analysis of local linkage disequilibrium (LD), marker–trait relationships and multilocus genotype patterns. In this study, a Python-based graphical user interface (GUI) was developed to integrate regional post-GWAS analyses and provide a button-driven alternative to command-line workflows. The platform includes PLINK-based regional marker extraction, LD calculation, LD decay visualisation, exploratory regional SNP–trait analysis, candidate LD region detection, graph-based LD clustering and pairwise genotype-combination analysis. As a proof of concept, the workflow was applied to publicly available genotype data from the Bengal and Assam Aus Panel and grain iron (Fe) concentration data from the same population. The analysis focused on a previously reported quantitative trait locus (QTL) located at approximately 29.28 megabase pairs (Mbp) on rice chromosome 7, near the candidate gene nicotianamine synthase 3 (*OsNAS3*). The GUI extracted 4894 single-nucleotide polymorphisms (SNPs) from a 1-Mbp interval and generated 11,973,171 pairwise LD comparisons. Exploratory regional SNP–trait analysis evaluated 4448 eligible SNPs. Because the GUI screening procedure did not account for population structure or relatedness, the regional SNPs were additionally evaluated using a mixed linear model incorporating five genome-wide principal components and a genomic kinship matrix. The adjusted analysis identified 133 Bonferroni-significant and 1635 Benjamini–Hochberg-significant SNPs, including marginal adjusted signals within the genomic neighbourhood of *OsNAS3*. At the specified clumping parameters, all 133 Bonferroni-significant SNPs were assigned to a single clump indexed by SNP 7:29285763, and no residual association remained significant after conditioning on this lead marker and correcting for multiple testing. Among 43,639 SNP pairs separated by no more than 1000 base pairs (bp), 22,952 had *r*^2^ ≥ 0.6. These strong-LD pairs were summarised as 116 candidate LD regions using interval-based aggregation and 339 graph-based LD clusters using connected-components analysis. Of the 22,952 strong-LD SNP pairs, 21,646 met the genotype-call eligibility criteria for pairwise genotype-combination analysis. Although exploratory pairwise analysis of variance (ANOVA) identified numerous corrected genotype–phenotype differences, nested-model comparisons provided no corrected evidence that any two-locus genotype combination explained additional phenotypic variation beyond its better constituent single-marker model. These findings reflect the extensive LD within the selected QTL interval and represent regional prioritisation rather than identification of causal variants. Overall, the GUI provides a reproducible framework for characterising GWAS-derived QTL intervals, regional LD patterns and genotype–phenotype relationships. Its broader applicability should be further evaluated using additional traits, populations, genomic regions and organisms with compatible PLINK genotype data.

## Introduction

Advances in DNA sequencing technologies and high-throughput genotyping have established genome-wide association studies (GWAS) as a powerful approach for dissecting the genetic architecture of complex traits [1–3]. GWAS detects associations between genetic markers and phenotypic traits and thereby facilitates the identification of quantitative trait loci (QTLs). However, QTL identification is usually followed by regional analyses to characterise linkage disequilibrium (LD), prioritise candidate variants and genes, and investigate the biological basis of the association [3, 4].

Local LD analysis helps determine whether nearby associated single-nucleotide polymorphisms (SNPs) are correlated and may represent the same underlying genetic signal [3, 5, 6]. For example, analysis of the complement factor H (*CFH*) region associated with age-related macular degeneration in humans showed that associated variants were in LD with the coding Y402H variant rs1061170 [7]. In rice, local LD supported interpretation of a grain-length association at the *GS3* locus containing the previously characterised functional variant snp_03_16733441 [8]. Therefore, region-specific LD analysis is crucial for interpreting individual GWAS signals, as genome-wide LD-decay estimates may not adequately capture the variation in recombination rates and LD patterns surrounding a particular QTL [5, 6].

In addition to LD analysis and single-marker SNP testing, multilocus analysis can provide further information about genetic variation within GWAS-associated regions [9, 10]. One form of multilocus analysis is haplotype analysis, which examines combinations of alleles at closely linked loci that are located on the same chromosome copy [11]. Haplotype analysis therefore requires chromosomal phase to be known or statistically estimated. By contrast, unphased genotype data can be used to compare observed multilocus genotype combinations without determining which alleles are located together on the same chromosome copy. For example, studies of coronary artery disease identified a strong association at the human chromosome 9p21 locus within an LD region containing disease-associated haplotypes [12, 13]. In rice, haplotype analysis of a candidate gene within a GWAS-identified locus revealed haplotypes associated with variation in shoot length [14]. These studies demonstrate the value of haplotype analysis, while exploratory assessment of LD patterns and unphased genotype combinations provide a distinct approach for prioritising regional genetic variation for further investigation.

Although regional LD and multilocus analyses can support the interpretation and refinement of GWAS signals, pinpointing the causal variants and genes underlying GWAS-identified QTLs remains a major challenge [3]. One principal difficulty is that the lead SNP identified by GWAS is often not causal but is correlated through LD with nearby variants. Consequently, a significant association signal may span a broad genomic region containing numerous correlated SNPs and multiple genes, making it difficult to identify the functional variant responsible for the observed phenotypic effect [2, 3, 6].

Addressing this challenge requires computational tools capable of extracting regional markers, calculating LD and supporting association analyses. PLINK is widely used for SNP data management, association testing and computation of LD statistics [15, 16]. Its computational efficiency and extensive analytical capabilities have made it a standard tool in genomic research. However, PLINK is primarily operated through a command-line interface, requiring familiarity with environments such as Linux, PowerShell or other terminal-based systems. This requirement may limit its accessibility among researchers with limited computational experience or those who prefer graphical user interfaces (GUIs) for genomic data analysis [16–18]. Graphical applications such as Haploview, TASSEL and Flapjack support aspects of SNP, LD and haplotype analysis or visualisation [19–21]. Nevertheless, regional post-GWAS investigation may require researchers to combine several programs or custom scripts for marker extraction, LD calculation, regional LD summarisation and exploratory genotype–phenotype analysis. Integrating these operations within one graphical workflow could improve accessibility and reproducibility.

To evaluate and demonstrate the utility of such an integrated GUI, accessible empirical datasets are required. Access to human datasets is often restricted by ethical, legal and privacy requirements [22, 23], whereas rice has extensive publicly available genomic resources, high-quality reference genomes and well-characterised diversity panels [24–28]. Rice therefore provides a suitable system for developing and evaluating regional genomic-analysis workflows.

In this study, a Python-based graphical user interface (GUI) was developed for exploratory regional analysis of GWAS-identified QTLs. The platform integrates regional marker extraction, pairwise LD calculation, LD-decay visualisation, exploratory regional SNP–trait analysis, candidate LD region detection, graph-based LD clustering and pairwise genotype-combination analysis. The workflow was demonstrated using publicly available genotype data from the Bengal and Assam Aus Panel and grain iron (Fe) concentration data from the same population [29, 30]. As a proof of concept, it was applied to a previously reported grain Fe QTL at approximately 29.28 megabase pairs (Mbp) on chromosome 7, near rice nicotianamine synthase 3 (*OsNAS3; LOC_Os07g48980*), a candidate gene involved in Fe transport and homeostasis [30–32].

## Methods

### Genotype and phenotype data

SNP genotype data were obtained from the Bengal and Assam Aus Panel (BAAP), a publicly available rice diversity panel deposited in the Harvard Dataverse repository (https://doi.org/10.7910/DVN/AUMTIH) [29]. The source dataset was generated by whole-genome sequencing and underwent quality control before deposition. Sequence reads were quality trimmed, aligned to the International Rice Genome Sequencing Project reference genome build 1.0 (IRGSP-1.0), and processed to remove duplicate reads. Following variant calling, biallelic SNPs were retained when the minor allele frequency (MAF) was >0.05 and genotype missingness was <0.10, resulting in 2,053,863 SNPs. Five accessions with sequencing depth below 1× and one accession with high heterozygosity were excluded, leaving 260 aus accessions [29].

This study used the quality-controlled PLINK dataset Aus_Admix_260_99_bi_01_005_beagle without applying additional marker or sample-level quality-control filters. The PLINK marker-information (MAP) file contained the chromosome and physical position of each marker, whereas the pedigree-format (PED) file contained the corresponding sample and genotype data [15, 16]. Phenotypic data for grain Fe concentration were obtained from Talukdar et al. [30]. Table S1 contained phenotype records for 282 accessions, of which 269 had non-missing grain Fe measurements. The extracted PED dataset contained 260 genotyped accessions. Sample identifiers were converted to character strings and stripped of leading and trailing whitespace before exact matching. A total of 248 genotyped accessions matched a phenotype record; nine of these had missing grain Fe measurements, leaving 239 accessions eligible for phenotype-based analyses. Twelve genotyped accessions had no corresponding phenotype record. No duplicate sample identifiers were detected in either dataset.

### Python GUI development

The GUI was implemented in Python using PyQt5 to provide the main window, input controls and button-driven workflow management [33, 34]. The application was organised into three modular scripts. The *initialise_funcs.py* script manages application initialisation, configuration-file input and output, default parameters and graphical resources. The *plinkUtilsGUI.py* script defines the main interface, including chromosome selection, genomic-coordinate entry, output-directory selection, root-filename specification and the buttons used to initiate each analysis. The *plink_funcs.py* script provides the computational backend, including input validation, PLINK command construction, regional marker extraction, pairwise LD calculation, LD-decay visualisation, exploratory regional SNP–trait analysis, candidate LD region detection, graph-based LD clustering and pairwise genotype-combination analysis.

**Fig. 1:**
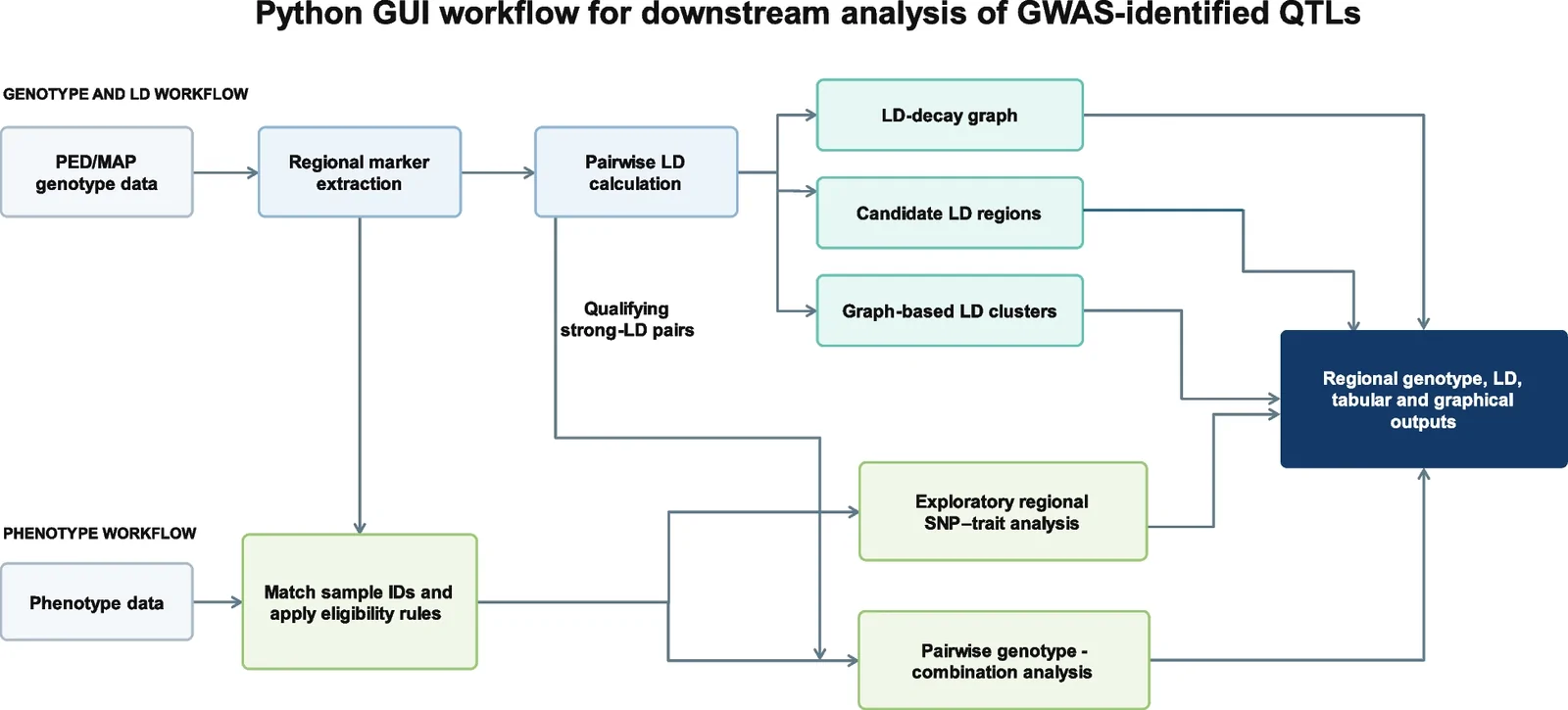
Overview of the Python GUI workflow for downstream analysis of GWAS-identified QTLs. The platform accepts PLINK PED/MAP genotype files and phenotype data. Regional marker extraction provides genotype data for pairwise linkage disequilibrium (LD) calculation and phenotype-based analyses after sample matching and application of genotype-eligibility criteria. Pairwise LD results are used for LD-decay visualisation, candidate LD region detection and graph-based LD clustering. Exploratory regional SNP–trait analysis uses matched regional genotype–phenotype data, whereas pairwise genotype-combination analysis additionally uses qualifying strong-LD SNP pairs. The workflow generates regional genotype, LD, tabular and graphical outputs

The application was developed and tested on Windows 11 using Python 3.12.11 and PLINK v2.0.0-a.7.6 (64-bit) [15, 16, 33, 34]. The required third-party Python packages were PyQt5 5.15.11, pandas 3.0.5, NumPy 2.5.2, Matplotlib 3.10.9, openpyxl 3.1.5 and psutil 7.2.2. The tested dependencies and their versions are specified in the requirements.txt file provided with the source code. After downloading or cloning the repository and retaining its directory structure, users can install the dependencies by running python -m pip install -r requirements.txt. The application can then be started by running python *plinkUtilsGUI.py* from the repository directory or by executing *plinkUtilsGUI.py* within a Python integrated development environment (IDE). Once launched, the graphical interface provides button-driven access to the analytical workflow summarised in Fig. 1.

The current GUI directly accepts PLINK text-format genotype datasets comprising a pedigree-format genotype file (PED) and a marker-map file (MAP). Other commonly used formats, including Variant Call Format (VCF) and the PLINK binary file set, are not read directly by the interface. The PLINK binary file set comprises a binary genotype table (BED), an extended marker-information file (BIM) and a family and sample-information file (FAM). These formats must first be converted to matching PED/MAP files using PLINK. For example, BED/BIM/FAM files can be converted using plink2 --bfile input_dataset --export ped --out converted_dataset, while VCF files can be converted using plink2 --vcf input_dataset.vcf --export ped --out converted_dataset. The resulting .ped and .map files must have the same root filename and be placed in the designated plink/map_ped directory before launching the analysis. Detailed conversion and input-placement instructions are provided in the repository README.

The GUI is independent of the software used to conduct the initial GWAS. Genomic intervals identified using PLINK, Genome Association and Prediction Integrated Tool (GAPIT), or other GWAS platforms can be analysed by manually entering the chromosome number and genomic coordinates into the interface [15, 16, 35]. The current version does not directly import GWAS summary-statistic files, GAPIT result tables or GAPIT-specific genotype objects. Instead, users must provide the underlying genotype data as matching PED and MAP files and specify the genomic interval of interest. Genotype data stored in other formats must first be converted to PED/MAP format using PLINK, as described above.

The source code, installation and execution instructions, input-format requirements and an example dataset are freely available at https://github.com/parthaomics/GWAS_Downstream_Analysis_Windows11. Although Python, PyQt5 and PLINK are available for Linux and macOS, the current release has been validated only on Windows 11.

### PLINK extract

Downstream analysis was initiated using a previously reported rice grain Fe QTL on chromosome 7, identified at approximately 29.28 Mbp using GWAS by Talukdar et al. [30]. As a proof of concept, this genomic region was analysed throughout the study to showcase the full functionality of the GUI. Marker extraction was performed by specifying chromosome 7 and defining the genomic interval from 28,780,000 to 29,780,000 bp, corresponding to a 1-Mbp target region. This interval can be adjusted by the user depending on the local LD decay profile, the required genomic resolution, the species under investigation and the specific objectives of the study [2, 36, 37]. Subsequently, the PLINK Extract function was applied to the input PED/MAP dataset to export markers within the selected chromosomal interval, generating a region-specific genotype subset for downstream LD, exploratory regional SNP–trait and pairwise genotype-combination analyses [15, 16]. Because the input dataset had previously undergone quality control, the PLINK Extract module applied only the specified chromosome and physical-position criteria; no additional MAF, missingness, heterozygosity or Hardy–Weinberg equilibrium filters were applied during regional extraction [29].

### LD decay analysis

Pairwise LD was estimated across the selected genomic interval (28.78–29.78 Mbp) using the LD-decay function, which invoked PLINK to calculate unphased pairwise *r*^2^. This statistic represents the squared correlation between allele counts at two loci and is one of the most widely used measures for quantifying LD in population-genetic data [5, 15, 16]. The analysis used a window of 99,999 variants, a maximum inter-marker distance of 1000 kb and no minimum *r*^2^ threshold. The resulting .vcor output provided the basis for LD-decay visualisation, candidate LD region detection, graph-based LD clustering and pairwise genotype-combination analysis [15, 16].

### LD decay graph

The LD Decay Graph function reads the pairwise LD output generated by PLINK in ‘.vcor’ (variant correlation) format during the LD decay analysis step. This file contains the genomic positions and unphased *r*^2^ values for all SNP pairs within the selected interval [15, 16]. For each SNP pair, physical distance was calculated as the absolute difference between the two marker positions (pos_b − pos_a). These distances were then grouped into 100-bp bins, and the mean *r*^2^ value was calculated for each bin. The resulting binned mean *r*^2^ values were plotted against physical distance to produce the LD decay curve. A 100-bp bin width was selected to retain fine-scale resolution across the 1-Mbp target interval while summarising the large number of individual pairwise LD observations. This value was used as an operational setting for the proof-of-concept analysis rather than as a universal biological threshold. The number of SNP pairs contributing to each bin was reported to support interpretation of the LD-decay profile. The graph was used to summarise how LD changed across the target region and to visualise the local LD structure.

### Exploratory regional SNP–trait analysis

Following identification of the chromosome 7 QTL in earlier work [30], the SNP–trait module was used as an exploratory regional screening procedure to rank variants within the predefined 1-Mbp interval. For each SNP, grain Fe concentration was compared among the observed genotype classes using one-way analysis of variance. A SNP was analysed only when every included genotype class was represented by at least three accessions. Accessions with missing genotype or phenotype data were excluded from the corresponding test. The analysis was intended to summarise and prioritise regional genotype–phenotype signals and was not treated as independent replication of the original GWAS or as evidence of causality. Population-structure, kinship and additional experimental covariates were not included in this exploratory regional analysis.

Prior to statistical analysis, missing and heterozygous genotype calls were coded as missing. Markers were eligible for analysis when homozygous genotype calls were available for at least 90% of the 239 phenotype-eligible accessions, corresponding to a minimum of 216 accessions. For each eligible marker, genotype classes containing fewer than three accessions were excluded, and the marker was analysed only when at least two genotype classes remained.

Because 4448 regional SNPs were tested, correction for multiple testing was performed using both Bonferroni family-wise error correction and the Benjamini–Hochberg false-discovery-rate (FDR) procedures [38, 39]. These complementary procedures were selected because Bonferroni correction provides stringent control of the family-wise error rate, whereas the Benjamini–Hochberg procedure controls the expected false-discovery rate and provides greater power when many hypotheses are tested. Bonferroni correction remains valid but can be conservative when tests are correlated, while neither procedure explicitly estimates the effective number of independent tests arising from regional LD. The Bonferroni threshold was *P* < 1.12 × 10^–5^, calculated as 0.05/4448 [38]. Benjamini–Hochberg-adjusted *q *values below 0.05 were considered significant under the FDR criterion [39]. Unadjusted *P* < 0.05 results were retained in the complete software output for exploratory use but were not interpreted as statistically significant findings.

To assess whether the regional SNP–trait signals persisted after accounting for population structure and relatedness, a complementary mixed linear model analysis was conducted outside the GUI using GAPIT v4.1 [35]. Genome-wide SNP data comprising 260 accessions and 2,053,863 variants were filtered using sample and marker missingness thresholds of 0.10 and a MAF > 0.05. Markers with observed heterozygosity greater than 0.10 were subsequently excluded, leaving 1,728,839 variants.

Linkage-disequilibrium pruning was performed using a 50-variant sliding window, a five-variant step and an *r*^2^ threshold of 0.20, retaining 62,813 approximately independent genome-wide markers. These markers were used to calculate the first ten principal components and a genomic kinship matrix in PLINK [15, 16]. The first five principal components were included as fixed covariates in the adjusted association model, while the kinship matrix was included as a random-effect covariance structure.

The adjusted analysis included the same 239 accessions with matched, non-missing grain Fe measurements and the 4448 regional SNPs that met the revised genotype-call eligibility criteria. Missing numerical genotype values were replaced with the marker mean before model fitting. Each regional SNP was tested using a mixed linear model containing the SNP effect, PC1–PC5 and the genomic kinship matrix. Multiple testing was addressed across the 4448 SNPs using a Bonferroni threshold of *P* < 1.12 × 10^−5^ and Benjamini–Hochberg-adjusted *q *values below 0.05. This analysis was used to evaluate the robustness of the exploratory GUI results and was not implemented as a GUI module.

LD-based clumping was subsequently applied to the adjusted mixed-model results using PLINK [15, 16]. Bonferroni-significant SNPs were eligible to act as index variants at *P* < 1.12 × 10^−5^. Variants within 1000 kb of an index variant and showing unphased LD of *r*^2^ ≥ 0.20 were assigned to the same clump. Index variants were selected sequentially from the smallest adjusted mixed-model *P *value. Clumping was used to summarise correlated regional associations and estimate the number of approximately independent signals; it was not interpreted as identification of causal variants.

A conditional mixed-model analysis was then performed to test for residual regional associations after accounting for the strongest adjusted SNP, 7:29285763. The lead-SNP dosage was added as a fixed covariate to the model containing PC1–PC5 and the genomic kinship matrix. The conditioning SNP was removed from the tested marker set, and the remaining 4447 regional SNPs were analysed. Bonferroni and Benjamini–Hochberg corrections were recalculated across the 4447 conditional tests.

### Candidate LD region detection

Pairwise LD patterns were used to identify candidate regions of local LD within the selected chromosome 7 interval. The Candidate LD Regions function read the pairwise LD output generated by PLINK and retained SNP pairs separated by no more than 1000 bp. Pairs with *r*^2^ ≥ 0.6 were classified as strong-LD pairs and sorted according to physical position. A custom interval-based procedure then aggregated spatially adjacent strong-LD pairs when the next pair started within 1000 bp of the current region boundary. Candidate-region boundaries were defined by the minimum and maximum physical positions of the constituent SNP pairs. The default thresholds of 1000 bp and *r*^2^ ≥ 0.6 were selected as operational criteria for summarising strong, short-range LD within the proof-of-concept interval. These settings were not intended to represent universal biological boundaries or an established haplotype-block definition.

For each candidate region, the software reported its genomic coordinates, physical span, number of constituent SNPs and mean, minimum and maximum pairwise *r*^2^. The complete set of SNP pairs separated by no more than 1000 bp was also exported, together with an indicator showing whether each pair met the *r*^2^ ≥ 0.6 threshold. This custom procedure was used as an exploratory spatial summary of local LD and was not intended to implement an established haplotype-block definition or infer phased haplotypes.

### Graph-based LD cluster detection

The graph-based LD clusters function provided a complementary network-based summary of the strong-LD relationships identified in the preceding analysis. SNPs were represented as nodes in an undirected graph, and an edge was created between two SNPs when they were located on the same chromosome, separated by no more than 1000 bp and had pairwise *r*^2^ ≥ 0.6. The resulting graph was partitioned into connected components using a union-find connected-components algorithm. Components containing at least two SNPs were reported as graph-based LD clusters.

For each cluster, the software recorded its minimum and maximum physical positions, genomic span, number of SNPs, number of qualifying LD edges and mean, minimum and maximum *r*^2^. Membership was determined by transitive connectivity; consequently, two SNPs could belong to the same cluster through intermediate SNPs even when they were not directly connected by a qualifying edge. The 1000-bp threshold applied to individual edges rather than to the total span of a connected cluster.

This graph-based procedure is a custom LD-network summarisation method. It does not implement the Dʹ confidence-interval method of Gabriel et al. [40], Haploview’s solid-spine algorithm or another established haplotype-block definition [19].

To assess the sensitivity of the LD summaries to parameter selection, candidate LD region detection and graph-based LD clustering were repeated using a 3 × 3 combination of maximum physical-distance thresholds (500, 1000 and 2000 bp) and minimum pairwise *r*^2^ thresholds (0.4, 0.6 and 0.8), yielding nine parameter combinations. For each combination, the numbers of qualifying SNP pairs, unique SNPs, candidate LD regions and graph-based LD clusters were recorded, together with the distributions of SNP counts and maximum genomic spans. The analyses also assessed whether the QTL-peak marker, 7:29285763, was assigned to an LD summary and whether the approximate genomic position of *OsNAS3* was encompassed by the resulting candidate regions or clusters.

### Pairwise genotype-combination analysis

Exploratory pairwise genotype-combination analysis was performed for same-chromosome SNP pairs separated by no more than 1000 bp and with pairwise *r*^2^ ≥ 0.6. Diploid genotypes were obtained from the PLINK PED file and combined across each pair of loci to construct an observed two-locus genotype code for every accession. Because chromosomal phase was not estimated in the workflow, these codes were interpreted as genotype combinations rather than chromosomal haplotypes.

Genotype and phenotype records were merged using sample identifiers, with grain Fe concentration used as the response variable. The same quality criteria were applied: each constituent marker was required to meet the 90% homozygous-call threshold, and each SNP pair was required to have joint homozygous genotype calls for at least 216 phenotype-eligible accessions. Missing and heterozygous calls were excluded. Grain Fe concentration was compared among the observed two-locus genotype-combination groups using ANOVA. Groups represented by fewer than three accessions were omitted, and a pair was analysed only when at least two eligible groups remained. Effect size was calculated as the ANOVA coefficient of determination *R*^2^, defined as the between-group sum of squares divided by the total sum of squares. Group sample sizes, means and standard deviations were reported with the ANOVA statistics. A total of 22,952 SNP pairs met the predefined LD and physical-distance criteria. After application of the individual-marker and joint homozygous-call requirements, 21,646 pairs were retained for statistical analysis. Multiple testing was addressed using Bonferroni and Benjamini–Hochberg false-discovery-rate corrections. The Bonferroni threshold was *P* < 2.31 × 10^−6^, calculated as 0.05/21,646, and Benjamini–Hochberg-adjusted *q *values below 0.05 met the FDR criterion. Unadjusted *P *values were retained in the complete output for descriptive purposes. The analysis was used for exploratory prioritisation within a previously identified QTL interval and was not interpreted as independent validation or evidence of causal genetic effects.

To determine whether a two-locus genotype combination explained phenotypic variation beyond its better constituent single marker, an additional nested-model analysis was performed. For each eligible SNP pair, the pairwise genotype-combination model and both constituent single-marker models were fitted to the same accessions. All models included PC1–PC5 as covariates. The two reduced models comprised PC1–PC5 and either SNP A or SNP B as a categorical genotype term. The full model comprised PC1–PC5 and the observed two-locus genotype-combination group. The constituent single-marker model with the smaller residual sum of squares was selected as the reduced model and compared with the full pairwise model using a partial *F*-test.

A pair was considered to provide estimable incremental information only when the full pairwise model had at least one additional degree of freedom relative to the selected single-marker model. Pairs for which the two-locus groups were completely determined by the individual constituent SNP were classified as having no estimable incremental information. Bonferroni and Benjamini–Hochberg corrections were applied across the valid nested comparisons. The incremental effect size was expressed as partial *R*^2^, calculated from the reduction in residual sum of squares between the reduced and full models.

For selected results, two-locus genotype-combination boxplots were generated by combining the pairwise-analysis output with the merged genotype–phenotype dataset. These plots were used to visualise phenotypic distributions among the observed genotype-combination groups. Genomic relatedness was not incorporated into the nested pairwise models; consequently, this analysis was used as an exploratory test of incremental two-locus information after adjustment for the first five principal components.

### Computational performance and output validation

Computational performance was evaluated on an HP EliteBook 840 G8 running Windows 11 Pro (build 26200), with an Intel Core i7-1165G7 processor, 32 GB random-access memory (RAM) and a 1 terabyte (TB) non-volatile memory express (NVMe) solid-state drive. The software environment is described in Sect. 2.2. Each operation was repeated three times using identical inputs and parameter settings.

Elapsed time was measured from initiation of an analytical operation until completion of output writing. Resident memory was sampled at a target interval of 0.1 s, and the largest simultaneously observed value was recorded as sampled peak resident memory. GUI measurements were performed in the IPython kernel and its descendant processes, whereas direct PLINK measurements represented the PLINK process.

Regional marker extraction and pairwise LD calculation were compared with direct PLINK execution using identical inputs and parameters. LD-decay graph generation was compared with direct invocation of the same Python plotting function. Output consistency was evaluated by comparing extracted MAP and PED records after whitespace normalisation and by comparing the record counts, file sizes and Secure Hash Algorithm 256-bit (SHA-256) checksums of the resulting .vcor files. The remaining workflow-specific modules were evaluated across three GUI runs, and successful completion was confirmed by generation of the expected tabular or graphical outputs.

## Results

### PLINK Extract

The PLINK Extract function was used to retrieve the target genomic interval on chromosome 7, defined by lower and upper coordinates of 28,780,000 bp and 29,780,000 bp, respectively. This extraction generated a region-specific PED/MAP dataset comprising 260 rice accessions and 4894 SNP markers within the selected 1-Mbp interval. The resulting dataset formed the basis for all subsequent analyses, including LD analysis, exploratory regional SNP–trait analysis, candidate LD region detection, graph-based LD clustering and pairwise genotype-combination analysis.

The MAP file contains marker information for each SNP, including the chromosome number, SNP identifier and physical position in base pairs along the chromosome. In contrast, the PED file contains sample-level genotype data, including individual identifiers and allele calls for each SNP marker across all accessions. The PED and MAP files together provide the genotype and marker position information required for subsequent downstream analyses.

### LD decay

Using the LD Decay function, pairwise unphased LD was calculated among all SNP markers within the extracted genomic interval. This analysis generated an LD matrix comprising 11,973,171 pairwise SNP comparisons, with *r*^2^ values ranging from 0 to 1 and physical distances between marker pairs ranging from 1 to 916,089 bp. The analysis produced a ‘.vcor’ output file containing pairwise LD statistics, which was subsequently used to generate the LD decay plot. The resulting LD data characterised the distribution of pairwise LD across the selected region and provided the basis for LD decay visualisation, candidate LD region detection, graph-based LD clustering and pairwise genotype-combination analysis. The numbers of SNP-pair comparisons and mean *r*^2^ values within successive 100-bp inter-marker-distance bins are provided in Table S2. Collectively across all 9104 distance bins, these comparisons account for a cumulative total of 11,973,171 pairwise LD records used to construct the regional LD-decay profile.

### LD decay graph

**Fig. 2:**
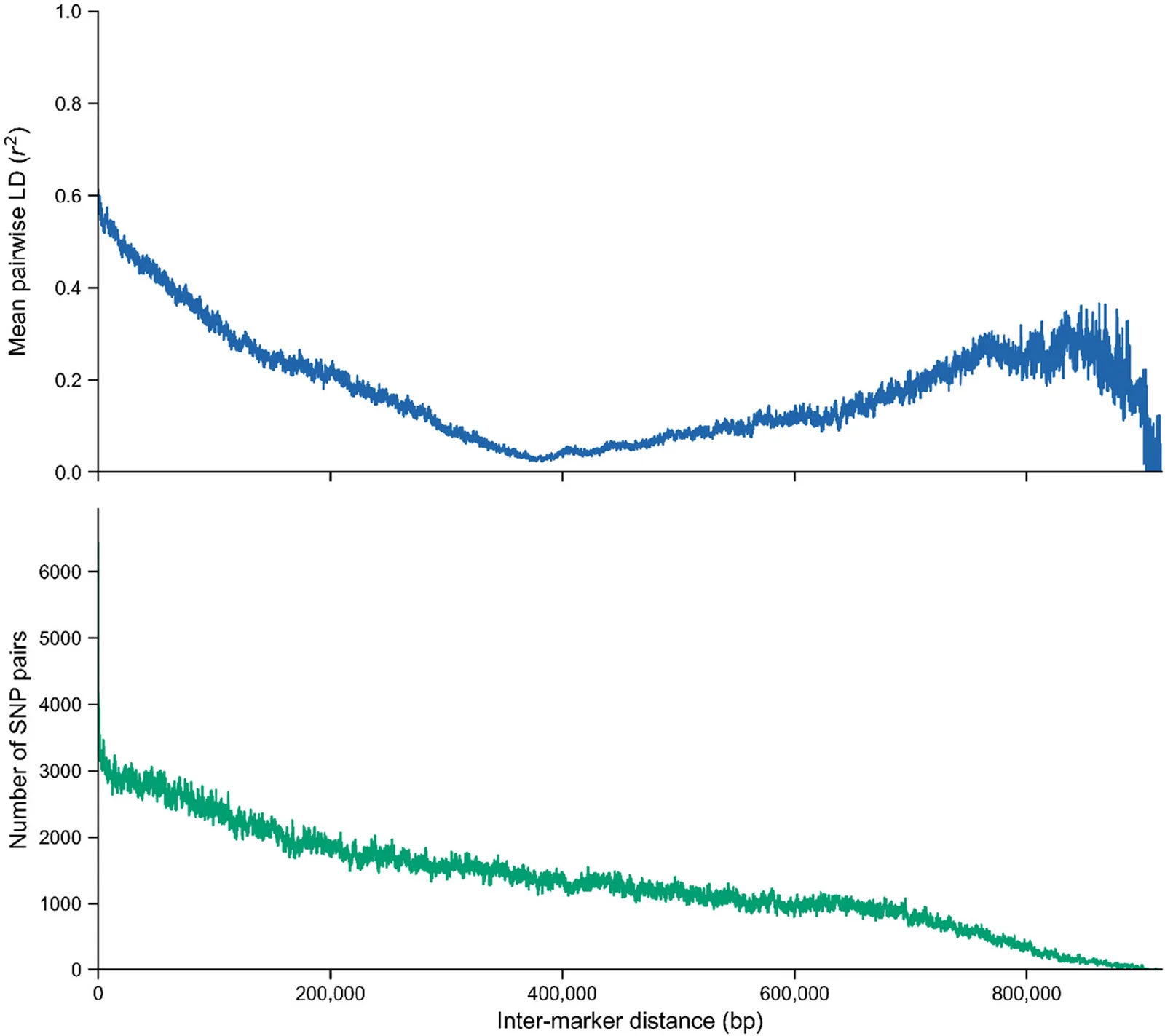
Linkage-disequilibrium decay and distribution of pairwise comparisons across inter-marker distances within the selected chromosome 7 interval. The horizontal axis shows the absolute physical distance between markers in base pairs. In the upper panel, the blue curve represents the mean pairwise linkage disequilibrium (*r*^2^) within successive 100-bp distance bins. In the lower panel, the green curve shows the number of SNP pairs contributing to each distance bin. The declining number of comparisons at larger inter-marker distances should be considered when interpreting variation in the corresponding mean *r*^2^ estimates

To visualise the relationship between LD and physical genomic distance across the extracted chromosome 7 interval, an LD-decay graph was generated using the pairwise LD statistics from the .vcor file (Fig. 2). The dual-panel plot outlines the rate of LD decay across the region by mapping absolute physical distance in base pairs (horizontal axis) against both mean pairwise *r*^2^ values (upper panel) and the corresponding number of SNP pairs contributing to each successive 100-bp distance bin (lower panel).

The LD decay curve revealed a clear distance-dependent pattern. Mean LD was highest among closely linked markers, with *r*^2^ values approaching 0.60 at short physical distances and declined rapidly as the distance between marker pairs increased. This decline continued to approximately 400 kbp, where mean LD reached a minimum of approximately *r*^2^ = 0.04, reflecting the progressive breakdown of allelic associations through historical recombination. Beyond this point, the curve exhibited a gradual increase in LD, with mean *r*^2^ values rising to approximately 0.30–0.35 between 750 and 850 kbp. Increased variability in *r*^2^ values was observed toward the distal end of the region, followed by a marked decline near the interval boundary. Overall, the observed LD profile indicated a heterogeneous LD landscape and provided the basis for subsequent candidate LD region detection and graph-based LD clustering. The increase in mean *r*^2^ at longer distances was interpreted cautiously because the number and distribution of SNP pairs contributing to the most distant bins may differ from those at shorter distances.

### Exploratory regional SNP–trait analysis

The merged genotype–phenotype output retained all 260 genotyped accessions, of which 239 contributed to the exploratory regional SNP–trait analysis. After missing and heterozygous genotype calls were treated as unavailable and the call-rate and genotype-class sample-size criteria were applied, 4448 SNPs were eligible for exploratory analysis. At the nominal *P* < 0.05 level, 2718 SNPs showed differences in grain Fe concentration among genotype classes. Following correction for 4448 regional tests, 2569 SNPs met the Bonferroni criterion and 2708 met the Benjamini–Hochberg false-discovery-rate criterion. The large number of retained signals was consistent with extensive LD across the selected interval and indicated that the exploratory analysis did not resolve the regional association to a single variant.

The strongest regional signal was observed for SNP 7:29613931 (*R*^2^ = 21.2%, *F* = 62.60, *P* = 1.03 × 10^−13^). This marker had eligible homozygous genotype calls for 234 accessions. The AA genotype group comprised 197 accessions with a mean grain Fe concentration of 11.46 mg kg^−1^, whereas the TT group comprised 37 accessions with a mean of 13.13 mg kg^−1^. Because the interval was selected following the original GWAS and contained extensive LD, this result was interpreted as regional prioritisation rather than independent confirmation of a causal variant. Complete single-marker results are provided in Table S3, and the merged genotype–phenotype dataset is provided in Table S4.

### Population-structure- and kinship-adjusted regional association analysis

**Fig. 3:**
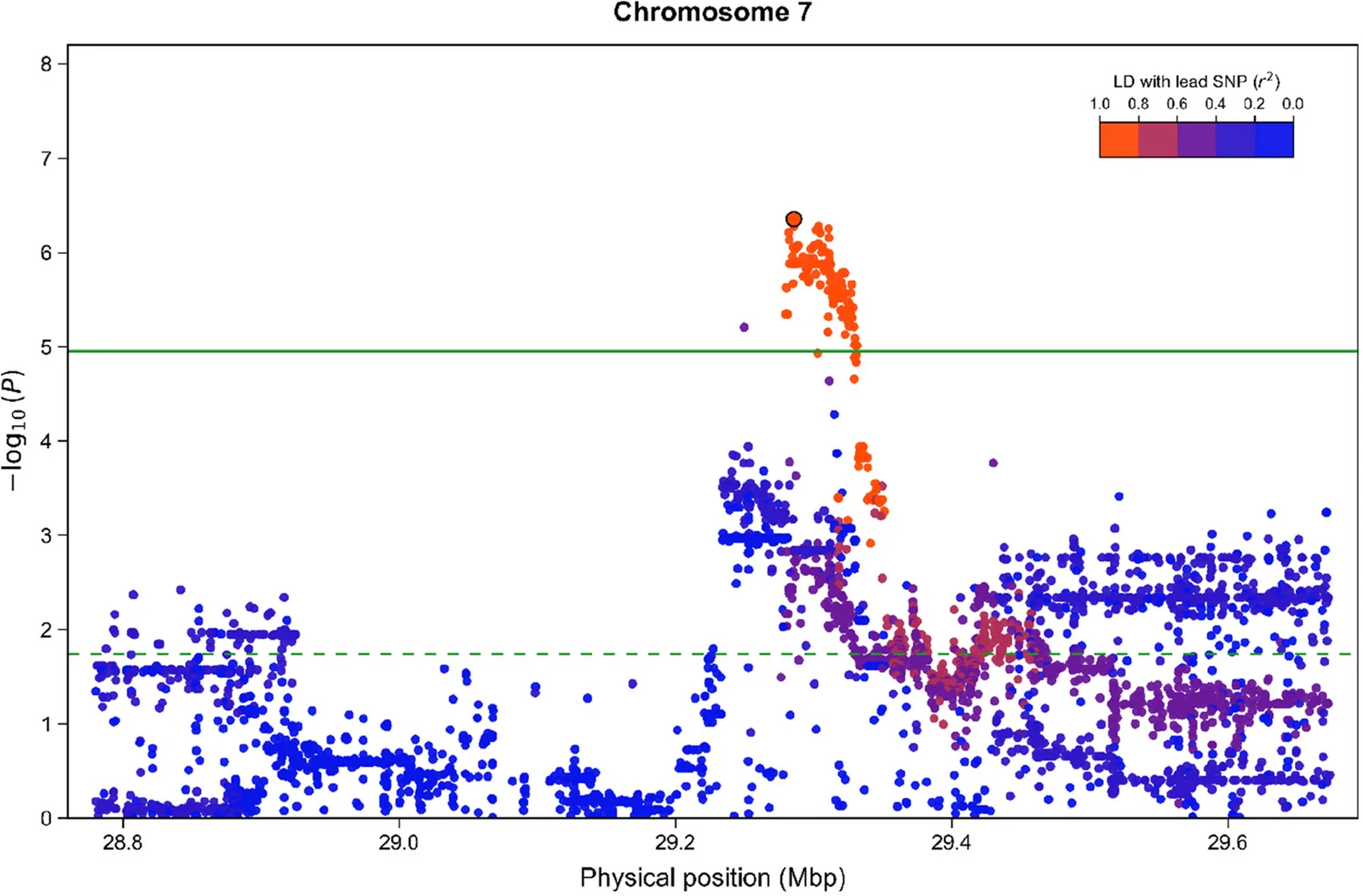
Regional association pattern after adjustment for population structure and genomic relatedness. Regional Manhattan plot of mixed-linear-model association results for 4448 eligible SNPs within the selected chromosome 7 interval. The horizontal axis shows physical position in megabase pairs, and the vertical axis shows −log_10_(*P*) for association with grain iron concentration. Each point represents one SNP, and point colour indicates pairwise linkage disequilibrium (*r*^2^) with the lead SNP, 7:29285763, with warmer colours indicating stronger LD. The solid and dashed green horizontal lines indicate the Bonferroni and Benjamini–Hochberg false-discovery-rate thresholds, respectively

The mixed linear model evaluated all 4448 eligible regional SNPs in the 239 phenotype-eligible accessions while accounting for five genome-wide principal components and genomic relatedness. Principal-component analysis showed the distribution of the 260 genotyped accessions across the first two principal components (Fig. S1). At the nominal *P* < 0.05 level, 2316 SNPs were associated with grain Fe concentration. After correction for 4448 regional tests, 133 SNPs met the Bonferroni criterion and 1635 met the Benjamini–Hochberg false-discovery-rate criterion. The regional Manhattan and quantile–quantile plots summarise the distribution of the adjusted association results in Fig. 3 and Fig. S2, respectively.

The strongest adjusted regional signal was observed for SNP 7:29285763 (*P* = 4.41 × 10^−7^, Benjamini–Hochberg-adjusted *q* = 1.10 × 10^−4^). Its estimated allelic effect was 0.484 mg kg^−1^, and inclusion of the SNP increased the model *R*^2^ by 8.63 percentage points. SNP 7:29320740, located near *OsNAS3*, also remained significant after adjustment for population structure, relatedness and multiple testing (*P* = 1.65 × 10^−6^, Bonferroni-adjusted *P* = 0.00736, Benjamini–Hochberg-adjusted *q* = 1.17 × 10^−4^). Its estimated effect was 0.461 mg kg^−1^, and its inclusion increased the model *R*^2^ by 7.73 percentage points. Complete mixed-model results are provided in Table S5.

LD clumping grouped all 133 Bonferroni-significant mixed-model SNPs into a single clump indexed by SNP 7:29285763 (Table S6). This result indicated that the corrected regional associations were not resolved into multiple approximately independent signals at the specified *r*^2^ and distance thresholds.

After SNP 7:29285763 was included as an additional covariate, 1371 of the remaining 4447 SNPs had nominal *P *values below 0.05, but none met the Bonferroni or Benjamini–Hochberg criteria. Complete conditional mixed-model results are provided in Table S7. The strongest residual result was observed for SNP 7:29641480 (*P* = 4.15 × 10^−4^, Benjamini–Hochberg-adjusted *q* = 0.122). The conditional result for SNP 7:29320740 near *OsNAS3* was not significant (*P* = 0.418, Benjamini–Hochberg-adjusted *q* = 0.512). These findings indicated that the marginally significant regional markers, including the marker near *OsNAS3*, did not provide evidence of association independent of the lead signal at 7:29285763.

### Candidate LD regions

Within the selected chromosome 7 interval, 43,639 SNP pairs were separated by no more than 1000 bp. Of these, 22,952 pairs met the strong-LD criterion of *r*^2^ ≥ 0.6. The interval-based aggregation procedure grouped the strong-LD pairs into 116 candidate LD regions containing 4458 unique SNPs.

The candidate regions contained a mean of 38.4 SNPs and a median of 11.5 SNPs, with region sizes ranging from 2 to 500 SNPs. Mean pairwise *r*^2^ within individual regions ranged from 0.6205 to 1.0000, with an overall mean of 0.8989 across the 116 regions. Sixty regions contained more than 10 SNPs, indicating extensive short-range LD within the selected interval. The largest candidate region extended from 29,284,519 to 29,354,950 bp, spanned 70,431 bp and contained 500 SNPs, with a mean pairwise *r*^2^ of 0.9314. Candidate-region summaries and the underlying pairwise LD records are provided in Table S8.

### Graph-based LD clusters

Connected-components analysis of the 22,952 qualifying LD edges identified 339 graph-based LD clusters comprising 4458 unique SNPs. The clusters contained a mean of 13.15 SNPs, and 93 clusters contained more than 10 SNPs. The largest cluster extended from 28,931,415 to 28,957,236 bp, spanned 25,821 bp and contained 201 SNPs connected by 1679 qualifying LD edges. Complete cluster assignments, genomic boundaries and LD summary statistics are provided in Table S9.

The graph-based procedure identified more groups than the interval-based procedure because spatially adjacent but disconnected LD networks were retained as separate clusters. In contrast, the candidate-region procedure could aggregate nearby strong-LD pairs into the same genomic interval without requiring the pairs to share a SNP. The two analyses therefore provided complementary spatial and network-based summaries of local LD rather than alternative formal definitions of haplotype blocks.

Threshold-sensitivity analysis showed that the numbers and genomic extents of the LD summaries varied with the selected physical-distance and *r*^2^ thresholds (Table S10). Across the nine parameter combinations, the number of candidate LD regions ranged from 33 to 320, while the number of graph-based LD clusters ranged from 98 to 652. At the thresholds used in the primary analysis—1000 bp and *r*^2^ ≥ 0.6—22,952 qualifying pairs involving 4458 unique SNPs were summarised as 116 candidate LD regions and 339 graph-based LD clusters. The QTL-peak marker 7:29285763 was assigned to candidate LD region 78 and graph-based LD cluster 126. The approximate genomic position of *OsNAS3* remained represented within the LD summaries across all nine parameter combinations. These results confirmed the presence of strong local LD while demonstrating that the numbers and boundaries of the resulting regions and clusters were parameter-dependent.

### Pairwise genotype-combination analysis

Pairwise genotype-combination analysis began with the 22,952 SNP pairs that met the predefined LD and physical-distance criteria. After applying the homozygous-call eligibility criteria, 21,646 pairs were tested. At the nominal *P* < 0.05 level, 13,881 pairs showed differences in grain Fe concentration among their eligible two-locus genotype-combination groups. Following correction for 21,646 tests, 13,145 pairs met the Bonferroni criterion and 13,665 met the Benjamini–Hochberg false-discovery-rate criterion. Complete results are provided in Table S11.

**Fig. 4:**
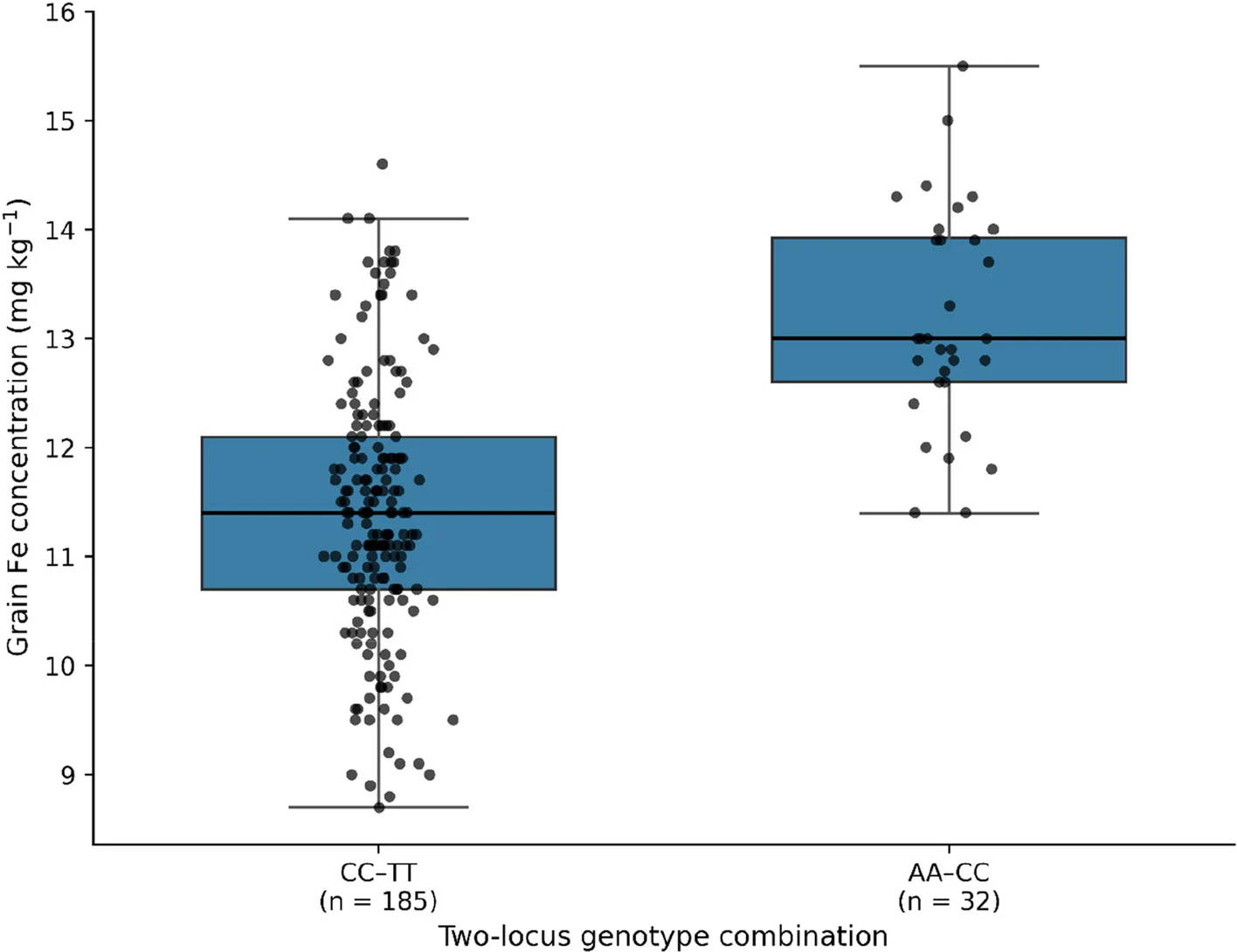
Distribution of grain Fe concentration among the two-locus genotype-combination groups defined by SNPs at 29,488,344 and 29,488,392 bp on chromosome 7. Missing and heterozygous genotype calls were excluded. The CC–TT and AA–CC groups contained 185 and 32 accessions, respectively. Boxes show the interquartile range, horizontal lines indicate the median, whiskers extend to 1.5 times the interquartile range, and points represent individual accessions

The strongest result involved markers 7:29488344 and 7:29488392, which were separated by 48 bp and had pairwise LD of *r*^2^ = 0.8289. Two eligible genotype-combination groups comprising 217 accessions were observed. The AA–CC group contained 32 accessions and had a mean grain Fe concentration of 13.17 mg kg^−1^, whereas the CC–TT group contained 185 accessions and had a mean of 11.40 mg kg^−1^. Genotype combination explained 22.8% of the phenotypic variation (*R*^2^ = 22.8%, *F* = 63.40, *P* = 9.65 × 10^−14^). The result met both the Bonferroni and Benjamini–Hochberg criteria. Phenotypic distributions across the two eligible genotype-combination groups are shown in Fig. 4. The two genotype-combination groups mapped directly onto the constituent single-marker genotype classes; therefore, this pair did not provide estimable incremental information beyond either individual marker.

The nested-model assessment showed that 16,517 of the 21,646 eligible pairs did not add an estimable model parameter beyond their better constituent single marker. For these pairs, the observed two-locus groups were completely determined by one constituent SNP. This category included the strongest exploratory pair, 7:29488344–7:29488392. Its two observed groups, AA–CC and CC–TT, corresponded directly to the genotype classes of either constituent marker. Consequently, the two-locus model was mathematically equivalent to a single-marker model, and an incremental partial *F*-test could not be performed.

The remaining 5129 pairs provided estimable nested comparisons, for which complete results are provided in Table S12. Of these, 856 had nominal incremental *P *values below 0.05, but none met the Bonferroni or Benjamini–Hochberg criterion. The strongest incremental result involved markers 7:29410909 and 7:29411889 and was evaluated in 230 accessions. Relative to the better constituent marker, 7:29410909, the two-locus model had an incremental partial *R*^2^ of 3.37% (*F* = 7.74, *P* = 0.00586, Benjamini–Hochberg-adjusted *q* = 0.295). Thus, the pairwise analysis did not identify corrected evidence that a two-locus genotype combination explained additional phenotypic variation beyond its better constituent SNP.

### Integrated regional analysis of the QTL and *OsNAS3*

The GUI-generated outputs were integrated to examine the relationships among the previously reported QTL peak, regional LD patterns and the genomic position of *OsNAS3*. Exploratory regional SNP–trait analysis identified corrected regional signals near the QTL peak at approximately 29.28 Mbp [30]. SNP 7:29285763 showed differences in grain Fe concentration between genotype groups (*R*^2^ = 19.7%, *F* = 57.81, *P* = 6.89 × 10^−13^) and met both the Bonferroni and Benjamini–Hochberg criteria (Table S3). This marker was also the strongest signal in the population-structure- and kinship-adjusted mixed linear model (*P* = 4.41 × 10^−7^, Benjamini–Hochberg-adjusted *q* = 1.10 × 10^−4^; Table S5). This SNP was located within candidate LD region 78, which extended from 29,284,519 to 29,354,950 bp, contained 500 SNPs and had a mean pairwise *r*^2^ of 0.9314 (Table S8). Graph-based analysis assigned the SNP to cluster 126, which extended from 29,284,519 to 29,291,101 bp, contained 28 SNPs and had a mean *r*^2^ of 0.9392 (Table S9). Exploratory pairwise genotype-combination analysis identified corrected differences among the genotype-combination groups defined by markers 7:29285763 and 7:29286293 (*R*^2^ = 20.3%, *F* = 59.63, *P* = 3.32 × 10^−13^; Table S11).

A similar pattern was observed in the genomic neighbourhood of *OsNAS3* (LOC_Os07g48980), located at approximately 29.32 Mbp [25]. SNP 7:29320740 showed corrected differences in grain Fe concentration between genotype groups (*R*^2^ = 18.5%, *F* = 53.6, *P* = 3.84 × 10^−12^; Table S3). This regional signal also remained significant in the complementary mixed linear model after adjustment for five genome-wide principal components and genomic relatedness (*P* = 1.65 × 10^−6^, Bonferroni-adjusted *P* = 0.00736 and Benjamini–Hochberg-adjusted *q* = 1.17 × 10^−4^; Table S5). This SNP was located within candidate LD region 78 and graph-based LD cluster 147. Cluster 147 extended from 29,310,477 to 29,333,986 bp, contained 192 SNPs and had a mean *r*^2^ of 0.9331 (Table S9). Exploratory pairwise-genotype-combination analysis also identified corrected differences among the groups defined by markers 7:29320740 and 7:29321473 (*R*^2^ = 18.5%, *F* = 53.6, *P* = 3.84 × 10^−12^; Table S11).

**Fig. 5:**
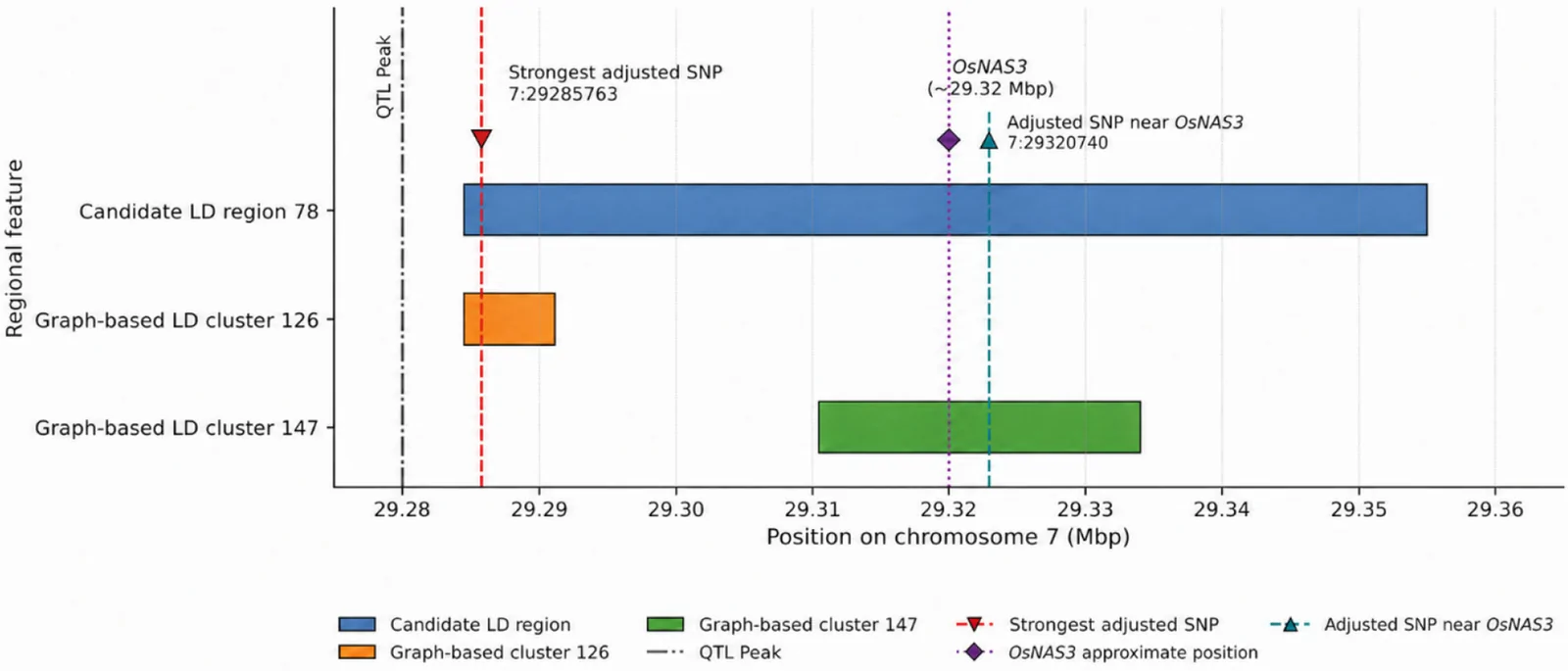
Regional relationship among the previously reported QTL peak, adjusted regional association signals, candidate LD region 78, graph-based LD clusters and *OsNAS3* on rice chromosome 7. The previously reported QTL peak is shown at its approximate position of 29.28 Mbp. SNP 7:29285763 was the strongest population-structure- and kinship-adjusted regional association, while SNP 7:29320740 represents the adjusted regional signal near *OsNAS3*. Candidate LD region 78 extended from 29,284,519 to 29,354,950 bp. Graph-based LD cluster 126 extended from 29,284,519 to 29,291,101 bp, whereas cluster 147 extended from 29,310,477 to 29,333,986 bp and encompassed the approximate genomic position of *OsNAS3*. Candidate LD regions and graph-based LD clusters represent threshold-dependent summaries of regional LD

Together, the exploratory GUI and adjusted mixed-model analyses identified a broad LD-correlated association signal spanning the previously reported QTL peak and the genomic neighbourhood of *OsNAS3* (Fig. 5). However, LD clumping assigned all 133 Bonferroni-significant markers to a single clump indexed by SNP 7:29285763, and no corrected association—including that of SNP 7:29320740 near *OsNAS3*—remained after conditioning on the lead SNP. These findings prioritise the broader genomic region for further investigation but do not establish an independent association with *OsNAS3* or identify a causal variant or gene.

### Computational performance and output validation

Runtime and sampled peak resident memory measurements for the GUI modules and their direct-execution comparators are summarised in Table 1. Each operation was repeated three times using identical input data and parameter settings.

**Table 1:** Runtime and sampled peak resident memory of the Python GUI workflow and direct-execution comparators. Values are medians, with ranges in parentheses, from three independent runs using identical input data and parameter settings. *MiB*, mebibytes; *LD*, linkage disequilibrium; *SNP*, single-nucleotide polymorphism

Operation	Execution mode	Repeats	Runtime, median (range), s	Sampled peak resident memory, median (range), MiB
Region extraction	Python GUI in Spyder	3	1.230 (1.221–1.301)	309.2 (300.9–320.7)
Region extraction	Direct PLINK in PowerShell	3	1.344 (1.279–1.348)	58.7 (48.6–87.4)
Pairwise LD calculation	Python GUI in Spyder	3	1.947 (1.942–2.002)	403.7 (401.6–420.5)
Pairwise LD calculation	Direct PLINK in PowerShell	3	2.180 (2.040–2.251)	149.4 (148.9–151.4)
LD decay graph generation	Python GUI in Spyder	3	31.855 (31.754–32.629)	1506.8 (1470.5–1512.4)
LD decay graph generation	Direct Python invocation	3	32.899 (32.194–34.002)	1457.9 (1454.0–1459.1)
Exploratory regional SNP–trait analysis	Python GUI in Spyder	3	4.635 (4.576–4.731)	428.5 (422.0–429.0)
Candidate LD region detection	Python GUI in Spyder	3	34.744 (34.212–35.239)	386.7 (386.4–387.1)
Graph-based LD cluster detection	Python GUI in Spyder	3	10.123 (10.036–10.262)	329.3 (327.6–334.6)
Pairwise genotype-combination analysis	Python GUI in Spyder	3	110.040 (109.775–110.305)	931.1 (930.0–931.8)

Regional marker extraction through the GUI had a median runtime of 1.230 s, compared with 1.344 s for direct PLINK execution. The corresponding median sampled peak resident memory values were 309.2 and 58.7 MiB, respectively. Both execution modes retained 4894 SNPs from 260 accessions, and the resulting MAP and PED records were identical. Pairwise LD calculation through the GUI had a median runtime of 1.947 s, compared with 2.180 s for direct PLINK execution, while the corresponding median sampled peak resident memory values were 403.7 and 149.4 MiB. Each run generated 11,973,171 LD records, and the .vcor files produced by the two execution modes had identical record counts, file sizes and SHA-256 checksums.

LD decay graph generation had a median runtime of 31.855 s through the GUI and 32.899 s through direct invocation of the same Python plotting function. Median sampled peak resident memory was 1506.8 MiB for GUI execution and 1457.9 MiB for direct Python execution. Both modes processed the complete LD dataset and generated the expected LD decay graph.

Among the four modules evaluated exclusively through the GUI, exploratory regional SNP–trait analysis had the shortest median runtime at 4.635 s, followed by graph-based LD cluster detection at 10.123 s and candidate LD region detection at 34.744 s. Pairwise genotype-combination analysis required the longest runtime, with a median of 110.040 s. Median sampled peak resident memory was 428.5 MiB for exploratory regional SNP–trait analysis, 329.3 MiB for graph-based LD cluster detection, 386.7 MiB for candidate LD region detection and 931.1 MiB for pairwise genotype-combination analysis. All four modules generated their expected outputs in each of the three runs.

## Discussion

### Integrated regional analysis platform

This study demonstrates that a Python-based GUI can integrate multiple steps in the downstream analysis of GWAS-identified QTLs to characterise associated intervals and prioritise regions for subsequent candidate-gene investigation. The workflow combines regional marker extraction, pairwise LD calculation, LD decay visualisation, exploratory regional SNP–trait analysis, candidate LD region detection, graph-based LD clustering and pairwise genotype-combination analysis. PLINK performs the principal genotype-processing and LD calculations, while the Python modules organise the inputs, execute the analyses and generate tabular and graphical outputs [15, 16]. Consolidating these operations within a single interface reduces the need to construct separate command-line and data-processing workflows and provides an accessible framework for exploratory regional analysis.

Existing tools provide complementary functionality for regional genetic analysis. LDBlockShow generates LD heatmaps and haplotype-block visualisations directly from Variant Call Format data, whereas geneHapR and CandiHap focus primarily on candidate-gene haplotype identification, haplotype statistics, phenotype comparison and visualisation [41–43]. By contrast, the present GUI integrates PLINK-based regional marker extraction and pairwise LD calculation with LD-decay visualisation, custom spatial and graph-based LD summaries, exploratory single-marker analysis and unphased two-locus genotype-combination analysis. The workflow does not replace these specialised tools and does not implement phased-haplotype inference or established haplotype-block definitions. Because the programs differ in their required inputs, analytical objectives and generated outputs, a direct computational comparison would not represent a like-for-like benchmark. The contribution of the present GUI is therefore its integration of complementary regional post-GWAS operations within a single button-driven workflow rather than analytical superiority over existing software.

The workflow is intended to assist with the characterisation and prioritisation of signals within pre-identified QTL intervals. Its outputs should not be interpreted as independent replication of the original GWAS, formal statistical fine-mapping or identification of causal variants. Instead, the GUI provides complementary summaries of regional association patterns, physical proximity and LD connectivity that can guide subsequent genetic and functional investigations.

For the directly comparable operations, GUI execution produced outputs identical to those generated through direct PLINK or Python execution and had similar runtimes under the tested configuration. GUI execution required more sampled resident memory than direct PLINK for regional marker extraction and pairwise LD calculation, reflecting the additional Python and graphical-interface processes included in the measured execution environment. LD-decay graph generation showed similar runtime and sampled peak resident memory under GUI and direct Python execution. These measurements demonstrate reproducible operation under the tested computing environment but should not be interpreted as universal performance benchmarks.

### Proof-of-concept QTL interval

The chromosome 7 interval selected to demonstrate the GUI workflow encompassed a previously reported QTL for grain Fe concentration, with an association peak at approximately 29.28 Mbp [30]. This interval encompasses the genomic neighbourhood of *OsNAS3*, which encodes a nicotianamine synthase involved in Fe transport and homeostasis in rice and is therefore a biologically relevant candidate gene for further investigation [31, 32].

The analysis conducted in this study revealed extensive LD and numerous correlated regional association signals across the selected interval. These findings indicate that the grain Fe QTL reported by Talukdar et al. was located within a region of substantial LD [30]. Such extensive LD makes it difficult to distinguish variants that contribute independently to phenotypic variation from variants that carry similar association signals because they are correlated with the same underlying genetic effect. The integrated results prioritise the genomic neighbourhood of *OsNAS3* for further investigation but do not establish that *OsNAS3*, or any individual variant within the interval, is causal.

### Exploratory regional association patterns

The exploratory GUI analysis identified numerous regional SNPs showing differences in grain Fe concentration among genotype classes. The large number of corrected signals was consistent with the extensive LD across the selected interval and should not be interpreted as evidence for thousands of independent genetic effects. Closely linked SNPs can carry similar association signals because their genotypes are correlated.

Because the GUI module uses one-way ANOVA and does not incorporate population structure or genomic relatedness, the regional markers were additionally evaluated using a mixed linear model containing five genome-wide principal components and a genomic kinship matrix. Adjustment substantially reduced the number of Bonferroni-significant markers from 2569 in the exploratory analysis to 133 in the mixed-model analysis. This reduction is consistent with population structure and genomic relatedness contributing to the unadjusted regional association pattern, although differences between the one-way ANOVA and mixed-model specifications may also have contributed.

Nevertheless, adjusted signals remained within the principal QTL interval. SNP 7:29285763 produced the strongest mixed-model result, and SNP 7:29320740 near *OsNAS3* remained significant under both Bonferroni and Benjamini–Hochberg correction. The persistence of these associations supports prioritisation of this genomic region after accounting for population structure and relatedness. However, because the regional analysis used the same population and phenotype as the preceding GWAS, these findings do not constitute independent replication. Higher-resolution statistical fine-mapping, validation in independent populations and functional investigation remain necessary to distinguish correlated signals and assess causal effects.

LD clumping and conditional analysis further clarified the structure of the regional association. All 133 Bonferroni-significant mixed-linear-model markers were assigned to one LD clump indexed by 7:29285763, and no corrected residual association remained after conditioning on this marker. The disappearance of the *OsNAS3*-near signal after conditioning indicates that it was not statistically independent of the lead regional signal. Nevertheless, because the interval contains extensive LD, these results do not establish that 7:29285763 is causal. The index SNP may instead tag one or more causal variants within the same correlated region. Higher-resolution mapping, independent populations and functional validation remain necessary to identify the underlying causal variation.

### Candidate LD regions, graph-based LD clusters and two-locus genotype patterns

Three complementary modules were used to characterise genetic variation within the selected chromosome 7 interval. Candidate LD region detection and graph-based LD clustering summarised the regional LD structure, whereas pairwise genotype-combination analysis examined phenotypic differences among observed two-locus genotype groups. The two LD modules analysed the same strong-LD relationships but applied different grouping rules. Candidate LD region detection aggregated spatially adjacent strong-LD pairs into broader genomic intervals, while graph-based clustering grouped SNPs into connected components based on direct or transitive LD connections. Consequently, nearby qualifying SNP pairs could be included in the same candidate LD region even when they did not share a SNP, whereas disconnected SNP networks remained separate in the graph-based analysis. This distinction explains why the interval-based procedure identified 116 candidate LD regions while the graph-based procedure identified 339 clusters.

The largest candidate LD region extended from 29,284,519 to 29,354,950 bp, contained 500 SNPs and had a mean pairwise *r*^2^ of 0.93. This region was located in the immediate vicinity of the approximate QTL peak reported by Talukdar et al. and encompassed the strongest adjusted regional SNP and the genomic neighbourhood of *OsNAS3* (Table S8) [25, 30]. Graph-based analysis resolved this broader region into smaller connected clusters, including one spanning 29,284,519–29,291,101 bp near the QTL peak and another spanning 29,310,477–29,333,986 bp that overlapped the *OsNAS3* region (Table S9) [25, 30]. These findings support prioritisation of the broader region containing *OsNAS3* for further investigation. However, the conditional analysis did not support an association near *OsNAS3* that was statistically independent of the lead regional signal, and functional validation is required before any causal role can be inferred.

Exploratory pairwise genotype-combination analysis identified numerous corrected differences in grain Fe concentration among observed two-locus genotype groups. However, the nested-model analysis showed that most pairs did not provide information beyond their better constituent single marker. For 16,517 pairs, the two-locus groups were completely determined by one constituent SNP, meaning that the pairwise and single-marker models were statistically equivalent. Among the remaining 5129 pairs with an evaluable incremental comparison, none remained significant after Bonferroni or Benjamini–Hochberg correction.

These findings indicate that the corrected pairwise ANOVA results primarily reflected the strong constituent single-marker associations and extensive LD within the selected interval rather than additional two-locus effects. The pairwise module remains useful for describing and visualising phenotypic distributions among observed genotype combinations, but significance from the exploratory pairwise ANOVA should not be interpreted as evidence that a SNP pair explains variation beyond its constituent markers. Tests of interaction or multilocus effects in other datasets will require adequate representation of recombinant genotype combinations and models appropriate to the population structure and relatedness of the study population.

### Practical utility and limitations

The principal strength of the GUI is its integration of regional post-GWAS operations within a reproducible, button-driven workflow. It provides direct access to PLINK-based marker extraction and LD calculation while producing LD plots, candidate-region summaries, graph-based clusters, exploratory association statistics and CSV, Excel and PNG outputs. The modular design also permits individual functions and thresholds to be modified for different datasets and research objectives. Although the GUI was initially developed for downstream analysis of GWAS-identified QTLs, it may also support exploratory regional analysis of user-defined genomic intervals. Provided that compatible genotype and phenotype data are available, the workflow can be used to examine regional LD patterns, identify candidate LD regions and graph-based LD clusters, and explore single-marker and unphased multilocus genotype–phenotype associations.

Several limitations should be considered. First, candidate LD regions and graph-based LD clusters depend on the selected *r*^2^ and physical-distance thresholds. Changing these thresholds may alter the number of regions or clusters identified, their genomic spans and the SNPs assigned to them. Graph-based clusters may also extend beyond the specified pairwise distance threshold through chains of qualifying LD connections. These outputs should therefore be interpreted as threshold-dependent summaries of regional LD structure rather than established haplotype blocks. This interpretation was supported by the sensitivity analysis, which showed that changing the physical-distance and *r*^2^ thresholds altered the numbers, genomic extents and SNP assignments of the resulting regions and clusters. The current interface applies predefined values for the LD-decay bin width, physical-distance threshold and *r*^2^ threshold. Although these parameters can be modified in the source code, they are not currently exposed as user-editable fields in the GUI. Incorporating direct parameter controls into the interface represents a priority for future development.

Second, the exploratory regional SNP and pairwise genotype-combination analyses used the same population and phenotype as the previously reported QTL analysis and therefore do not provide independent validation [30]. Population structure and genomic relatedness were incorporated into the complementary single-marker mixed model, whereas the GUI-based one-way ANOVA and pairwise genotype-combination analyses did not include these controls. The nested-model analysis incorporated PC1–PC5 to account for population stratification but did not include the genomic kinship matrix. Consequently, the pairwise results should be interpreted as exploratory. Extensive LD also resulted in substantial correlation among the statistical tests; therefore, the number of significant adjusted *P *values should not be interpreted as the number of independent genetic effects. Bonferroni and Benjamini–Hochberg corrections were used because they are transparent, reproducible and widely applied, but neither procedure explicitly models the dependence structure generated by LD. Permutation-based thresholds or corrections based on the effective number of independent markers could provide complementary multiplicity control in future implementations. The number and composition of LD clumps also depend on the selected *r*^2^ and physical-distance thresholds. The single-clump result should therefore be interpreted as a threshold-dependent summary rather than definitive evidence for a single causal variant.

Third, the pairwise-genotype-combination module was restricted to physically proximal SNP pairs separated by no more than 1000 bp and having *r*^2^ ≥ 0.6. It was designed to describe genotype combinations among strongly correlated regional markers rather than to perform an exhaustive search for statistical interactions. Potentially informative combinations involving markers in weaker LD or separated by greater physical distances were therefore not evaluated. Future implementations should permit user-defined LD and distance thresholds and support interaction-specific models across a broader range of marker pairs.

Fourth, the workflow combined diploid genotype calls at pairs of loci without estimating chromosomal phase. The resulting codes therefore represent observed two-locus genotype combinations rather than confirmed haplotypes.

Fifth, the current GUI directly accepts PLINK PED/MAP genotype files and has been validated on Windows 11. It does not directly import GAPIT association-result tables or GAPIT-specific genotype objects. Nevertheless, genomic intervals identified through GAPIT or other GWAS software can be analysed after users enter the chromosome and genomic coordinates and convert the underlying genotype data to PED/MAP format. Direct support for additional genotype and GWAS-output formats and validation on Linux and macOS represent priorities for future development. Candidate-gene coordinates were incorporated manually in the present proof-of-concept analysis because the GUI does not currently retrieve gene annotations from external databases. Automated annotation retrieval and additional regional visualisations, including LD heatmaps, would improve the interpretation of future analyses.

Finally, the present demonstration was restricted to one rice QTL and one trait. Evaluation using additional traits, genomic regions, populations and species will be required to establish the broader applicability of the workflow.

## Conclusion

The Python GUI provides an integrated and reproducible workflow for exploratory regional analysis of GWAS-derived QTLs, including marker extraction, pairwise LD calculation, LD-decay visualisation, exploratory SNP–trait analysis, candidate LD region detection, graph-based LD clustering and pairwise genotype-combination analysis. The exploratory pairwise module described phenotypic differences among observed two-locus genotype combinations; however, nested-model analysis found no corrected evidence that these combinations explained additional phenotypic variation beyond their better constituent single-marker models. Application to a previously identified rice grain Fe QTL revealed extensive LD and regional genotype–phenotype signals within the chromosome 7 interval encompassing the genomic neighbourhood of *OsNAS3*. Complementary mixed-model analysis identified adjusted regional associations near the QTL peak and *OsNAS3*. However, LD clumping grouped all Bonferroni-significant markers into a single clump, and no corrected residual association, including the signal near *OsNAS3*, remained after conditioning on the lead SNP. These findings are consistent with a principal LD-correlated regional signal under the selected clumping parameters, but they do not provide independent validation or identify a causal variant or gene. The GUI provides a button-driven framework for exploratory post-GWAS characterisation. Its broader applicability should be evaluated using additional traits, genomic regions, populations and PLINK-supported organisms for which compatible genotype and phenotype data are available.

## Supplementary Information

**Additional file 1: Table S1**. Grain iron phenotype records for 282 accessions.

**Additional file 2: Table S2**. Numbers of SNP pairs and mean *r*^2^ values within 100-bp inter-marker-distance bins used to construct the LD-decay profile.

**Additional file 3: Table S3**. Exploratory regional SNP-trait analysis.

**Additional file 4: Table S4**. Merged regional genotype–phenotype dataset containing 260 genotyped accessions.

**Additional file 5: Table S5**. Population-structure- and kinship-adjusted regional SNP–trait association results.

**Additional file 6: Table S6**. LD clumping of population-structure- and kinship-adjusted regional association results.

**Additional file 7: Table S7**. Conditional mixed-model analysis of regional SNP–trait associations.

**Additional file 8: Table S8**. Candidate LD regions and pairwise LD records within 1000 bp.

**Additional file 9: Table S9**. Graph-based LD clusters.

**Additional file 10: Table S10**. Sensitivity of candidate LD region detection and graph-based LD clustering to the physical-distance and *r*^2^ thresholds.

**Additional file 11: Table S11**. Exploratory pairwise genotype-combination analysis.

**Additional file 12: Table S12**. Nested-model results for the 5129 SNP pairs that provided estimable comparisons between the two-locus genotype-combination model and the better constituent single-marker model.

**Additional file 13: Figure S1**. PC1 versus PC2 plot for the 260 genotyped accessions. Figure S2 Quantile–quantile plot from the GAPIT mixed-model analysis.

## Data Availability

The genotype data analysed in this study are publicly available through the Harvard Dataverse repository at https://doi.org/10.7910/DVN/AUMTIH. The phenotype data and derived results supporting the findings are provided within the article and its Supplementary Information.
