# Supplementary material for: A Python GUI for integrated regional analysis of GWAS-identified QTLs
Source: Genomics Inform. 2026 Nov 10;24:27. doi: 10.1186/s44342-026-00091-6 (PMC13749646; doi:10.1186/s44342-026-00091-6)
Supplement: Supplementary file 1 [file 44342_2026_91_MOESM13_ESM.docx]

**Supplementary Figures:**

Figure S1: Principal-component analysis of the 260 genotyped rice accessions based on 62,813 linkage-disequilibrium-pruned genome-wide single-nucleotide polymorphisms. Each point represents one accession. The horizontal and vertical axes show the scores for the first and second principal components (PC1 and PC2), respectively. Blue circles represent the other accessions, whereas red circles with black outlines identify the seven accessions highlighted by the heterozygosity diagnostic. The sample identifiers 26, 35, 53, 61, 153, 181 and 275 are shown beside the corresponding red points. These accessions are highlighted for diagnostic inspection; the colours do not denote phenotype classes or association-test results.

Figure S2: Quantile-quantile plot of association-test P-values from the regional mixed linear model for grain iron concentration. The model tested 4,448 eligible single-nucleotide polymorphisms and included the first five genome-wide principal components and the genomic kinship matrix to account for population structure and relatedness. Each blue open circle represents one tested marker. The horizontal axis shows the expected −log10(*P*) values under the null hypothesis, and the vertical axis shows the corresponding observed −log10(*P*) values. The red diagonal line represents equality between observed and expected values, and the grey band shows the confidence envelope expected under the null distribution. Points above the diagonal represent smaller observed *P*-values than expected under the null.
